# Supplementary material for: Whole genome de novo assemblies of three divergent strains of rice, Oryza sativa, document novel gene space of aus and indica
Source: Genome Biol. 2014 Dec 3;15(11):506. doi: 10.1186/s13059-014-0506-z (PMC4268812; doi:10.1186/s13059-014-0506-z)
Supplement: Additional file 1: — Contains the supplementary tables and figures described in the manuscript. [file 13059_2014_506_MOESM1_ESM.pdf]

Whole genome de novo assemblies of three divergent strains of rice (*O. sativa*) documents novel gene space of *aus* and *indica*

Supplementary Tables and Figures

Table of contents

Supplementary Table 1. Maker input Sequences
Supplementary Table 2. Gene summary statistics of MAKER-P annotation and comparison to MSU and RAP-DB rice annotations of IRGSP1.0.
Supplementary Table 3. Alternate Nipponbare assemblies
Supplementary Table 4a. Nipponbare-specific PCR Validated regions
Supplementary Table 4b. IR64-specific PCR validated regions
Supplementary Table 4c. DJ123-specific PCR validated regions
Supplementary Table 5. Scaffold sizes containing the regions of interest
Supplementary Table 6. Primers for regions of interest
Supplementary Table 7. Distance from gene to nearest scaffold gap.
Supplementary Figure 1. K-mer frequency distributions for the three genomes.
Supplementary Figure 2. K-mer coverage of the S5 locus.
Supplementary Figure 3. Multiple alignment of S5 Hybrid Sterility Locus: Indel Region
Supplementary Figure 4. Multiple alignment of S5 Hybrid Sterility Locus: SNP region
Supplementary Figure 5. K-mer coverage across the Sub1B gene.
Supplementary Figure 6. K-mer coverage across the Sub1C gene.
Supplementary Figure 7. K-mer coverage across the entire LRK region.
Supplementary Figure 8. K-mer coverage across the LRK1 gene
Supplementary Figure 9. K-mer coverage across the Pstol1 gene
Supplementary Figure 10. K-mer coverage across the entire Pup1 region in Kasalath
Supplementary Figure 11. Multiple alignment of Pstol1 sequence fragment.

Supplementary Table 1. Maker input Sequences

Sequence evidence used for protein-coding gene annotation by MAKER-P

Evidence	Sequence Count
Oryza EST (NCBI)	1,319,326
Oryza FL_CDNA & mRNA (NCBI)	61,203
Nipponbare annotated CDS (non-redundant IRGSP-1.0 + MSU7.0: Kawahara e al. 2013)	73,621
93-11 annotated CDS (Gao et al. 2013)	37,573
PA64s annotated CDS (Gao et al. 2013)	34,690
Nipponbare annotated proteins, non-redundant IRGSP-1.0 + MSU7.0 (Kawahar et al. 2013)	67,725
93-11 annotated proteins (Gao et al. 2013)	37,573
PA64s annotated proteins (Gao et al. 2013)	34,690

Supplementary Table 2. Gene summary statistics of MAKER-P annotation and comparison to MSU and RAP-DB rice annotations of IRGSP1.0.

Assembler / Parameters	DJ123 MAKER	IR64 MAKER	Nipponbare MAKER	IRGSP1.0 MSU	IRGSP1.0 RAP-DB
Gene count (protein-coding)	37812	37758	39083	39049	35472
Median gene length (nt)	2285	2275	2224	2188	2458
Average transcripts/gene	1.36	1.35	1.34	1.26	1.18
Median coding length (nt)*	873	873	846	849	803
Median peptide length (aa)*	291	291	282	283	268
Median exon length (nt)*	157	158	157	165	177
Median intron length (nt)*	185	186	188	174	155
Average exons/transcript*	4.9	4.8	4.8	4.3	4.4
Percent genes single-exon	20.7	20.6	20.7	24.9	28.8
*Based on single representative transcript per gene having longest coding sequence					

Supplementary Table 3. Alternate Nipponbare assemblies

Note, the SOAPdenovo assemblies used Quake to error correct the reads before assembly (indicated with a *), while ALLPATHS-LG and SGA have integrated error correction packages so used the raw reads directly.

**Assembler / Parameters**	**Libraries**	**Span (Mbp) Bases (Mbp)**	**Scaffold N50 (kbp)**	**Contig N50 (kbp)**
**ALLPATHS-LG**				
+ MIN_CONTIG=300	180bp frag<br>2.1kbp jump<br>4.8kbp jump	355.6<br>322.5	213.7	21.5
+ MIN_CONTIG=300	180bp frag<br>1.8kbp jump<br>2.1kbp jump	357.6<br>317.2	98.7	20.8
**SOAPdenovo**				
+ K=33	180bp frag*<br>2.1kbp jump*<br>4.8kbp jump*	364.9<br>263.3	5.5	0.6
+ K=35	“	368.7<br>272.8	5.9	0.8
+ K=37	“	366.3<br>281.9	6.2	1.0
+ K=39	“	368.1<br>289.7	6.6	1.2
+ K=41	“	368.7<br>296.4	7.0	1.4
+ K=43	“	359.1<br>302.7	5.6	1.7
+ K=45	“	359.8<br>307.5	5.7	1.9

Assembler / Parameters	Libraries	Span (Mbp) Bases (Mbp)	Scaffold N50 (kbp)	Contig N50 (kbp)
**SGA**				
+ K=71	180bp frag<br>2.1kbp jump<br>4.8kbp jump	380.3<br>512.1	13.6	1.2
+ K=73	“	379.3<br>498.4	13.8	1.5
+ K=75	“	378.3<br>485.5	13.9	1.7
+ K=77	“	376.7<br>472.9	13.9	2.0

Supplementary Table 4a. Nipponbare-specific PCR Validated regions

Note the ordering and gene status has slightly changed since the analysis was finalized for publication.

#	Scaffold / Coordinates	Forward Primer / Reverse Primer	Notes
1	scaffold_45<br>198303 - 211765	AACATAGCGGGGAAGGCCTT<br>GACGCCAGTCTAGGTCCCAC	
2	scaffold_450<br>64623 - 76993	TGTCCTCCACGTGCCTGTTC<br>TTCACAAGTGGACCGCTCGT	
3	scaffold_849<br>41087 - 53325	TACGCCGCCGTCAATACCAG<br>TGGACGAGAGGAAGAGGGGT	
4	scaffold_139<br>54757 - 66719	GGCACCTCGCATCTCAAGGT<br>CGGGCACAATTCCGACTACAC	
5	scaffold_37<br>99481 - 111372	TTGGGCCGTAAGCTTGAACC<br>CATCATGTGCCTTGTGTACGTG	
6	scaffold_995<br>40472 - 51224	TCTCGCTGGACGGTCTCTGA<br>ACCTTCCCTTGTTGATGCCCT	Currently 8th longest
7	scaffold_18<br>671704 - 681347	GTGCCACACTTGCTGATGGC<br>ACCATCAGTTTGTTTTCGGCCC	Currently 9th longest
8	scaffold_290<br>184750 - 192614	GGAACGCTGAGGCACACAAG<br>GACGTTGTTGCCAGGCTCAG	No longer intersects an exon
9	scaffold_77<br>448149 - 458404	CTGAACCACATGACCGCTGC<br>GATCGTCGGCTTGTCGGAGA	Currently 10th longest
10	scaffold_2377<br>14202 - 21834	TTTTTCCGCCGGCCAAAACG<br>TGGGCCGGAGTAAAACAATCA	Currently 11th longest

Supplementary Table 4b. IR64-specific PCR validated regions

Note the ordering and gene status has slightly changed since the analysis was finalized for publication.

#	Scaffold / Coordinates	Forward Primer / Reverse Primer	Notes
1	scaffold_918<br>12095 - 27494	GGCATTAGCACAGGCAGCAG<br>CAATTGAAAGGCCACTCACCCT	
2	scaffold_712<br>115731 - 129191	AGTATTCAGCTCTGTGGCAGCA<br>TGGATCGACACAGCTCCGTG	
3	scaffold_1091<br>78493 - 90663	GGTGCGTGTTGGATGATGCT<br>ACTGATTGGACAAGGGCGGC	
4	scaffold_408<br>2395 - 14990	GCGCTTGAGTTGGGATGCTA<br>ACGGCATTAGCAGGGGACAA	
5	scaffold_299<br>284265 - 296829	CCCTCCTTTGTGACAGGCCA<br>GCGGCAACAGACTCGTTATCG	
6	scaffold_479<br>190704 - 202637	CACGAGTTGCAACTGCCAGT<br>ACATGTGCCGATCCCATGGA	Currently 7th longest
7	scaffold_382<br>149495 - 161037	ACAGGACGGGACTGTTCGTC<br>TTGAGCCCTTCATGCACCCT	Currently 8th longest
8	scaffold_1261<br>47601 - 59036	GCCCATACACCGTCATGGGT<br>GGACAGCGTGGTGTACAGAGA	Currently 9th longest
9	scaffold_201<br>190458 - 201884	CCATATGCCGGCCAGGATCT<br>TAGTGTGGCACATCGCAACC	Currently 10th longest
10	scaffold_977<br>3058 - 14164	GTTGTCGTCGCATCCGTGTC<br>CCATGGATCAACCCGGTGTG	Currently 11th longest

Supplementary Table 4c. DJ123-specific PCR validated regions

Note the ordering and gene status has slightly changed since the analysis was finalized for publication.

Num	Scaffold / Coordinates	Forward Primer / Reverse Primer	Notes
1	scaffold_1266<br>34254 - 47074	CTGCCACAAGCCTCCCAATT<br>CCCAGGGCTCCTTATGCGAT	No longer intersects a gene
2	scafold_903<br>70491 - 82898	TCCGCAGCATAGAAGGCCC<br>GCCCATAGATGCGCCATCCA	
3	scaffold_1392<br>10086 - 22243	ACAGCCACTGCCACATGTGA<br>TGGGCTGATCGATCATGCGA	
4	scaffold_185<br>381192 - 292627	AACCGAGCGACTGTAGCCAC<br>TGATGCTCTTGAGGGCGACA	
5	scaffold_318<br>70856 - 82242	TTGACCATGGGCAAGACTGG<br>GCTCATTGGACAAGGCGGTT	
6	scafold_289<br>28477 - 49393	TATCAGCGTCGACCTGGTGG<br>ACAAGCTGCCTCACCGATGT	Currently 7th longest
7	scaffold_328<br>55971 - 66840	TGGGCCCAATCAGATGCCAT<br>GAGCGACCCCTTAGGCCTTC	Currently 8th longest
8	scaffold_92<br>124892 - 135721	TGACGGAGCTGCTGAAGGAG<br>TTGACAAGGCAGCGACGGAT	Currently 9th longest
9	scaffold_82<br>177274 - 187967	TGGACATTGTGGTGCAGCCA<br>ACCTGCTCCAAACCAGTCGA	Currently 10th longest
10	scaffold_132<br>88873 - 99280	CCACCCACCACTCGCACTAG<br>AAGCAACACGGTGTCGGAGA	Currently 11th longest

Supplementary Table 5. Scaffold sizes containing the regions of interest

Region	Nipponbare	IR64	DJ123
S5	279.2 Kb	542.2 Kb	499.5 Kb
Sub1	49.6 Kb	520 Kb	299.5 Kb
LRK	96.4 Kb	210.2 Kb	299 Kb
Pup1	50.5 Kb<br>36.5 Kb	217.8 Kb	114.8 Kb<br>69.2 Kb

Supplementary Table 6. Primers for regions of interest

Primers for Sanger sequencing to confirm polymorphisms in S5 region

S5-Fwd1	TGCCCCTGAGCAAGCAAGAA
S5-Fwd2	CCTACGTTTGACTGCCTGCC
S5-Fwd3	GTTCGGGTGCAGCATGGATG
S5-Rev1	ACTACTACACGCGGCTTCGG
S5-Rev2	TGGCGCCTTGAGAGTTCACA
S5-Rev3	GTGTAGCGCGGGAGAAGACT

Primers for Sanger sequencing validation of *Pstol1* SNPs

Pstol-Fwd1	ATGGCCGTGAGATAGCCGTC
Pstol-Rev1	AAGCCCTTTTGGTGGCAACG

Supplementary Table 7. Distance from gene to nearest scaffold gap.

For each annotated gene in the 3 assemblies, we computed the distance to the nearest scaffold gap or to the end of scaffold from the 5' and 3' ends of each gene. We observe the strain-specific genes to be modestly closer to scaffold gaps than genes shared in all three strains: median of 12-14kbp for shared vs 8-10kbp for strain-specific. Across all sets less than 12% of the genes have less than 1000bp of flanking sequence. In a minority of cases the gene model would span across a scaffold gap, giving a zero or negative flanking distance.

Analysis Set	***Gene count***	***Median 5' flank length***	***Median 3' flank length***	***Num. Genes <1000bp 5' flank (%)***	***Num. Genes <1000bp 3' flank (%)***
IR64/ Nipponare/ DJ123	109,865	12,947	14,059	7,896 (7.2)	6,585 (6.0)
IR64/ DJ123	1,209	8,451	8,976	114 (9.4)	97 (8.0)
DJ123/ Nipponbare	1,156	8,425	9,086	108 (9.3)	98 (8.5)
IR64/ Nipponbare	959	6,858	7,826	124 (12.9)	101 (10.5)
IR64	381	9,112	8,049	36 (9.4)	42 (11.0)
DJ123	297	9,911	9,808	24 (8.1)	21 (7.1)
Nipponbare	786	8,980	9,023	94 (12.0)	74 (9.4)

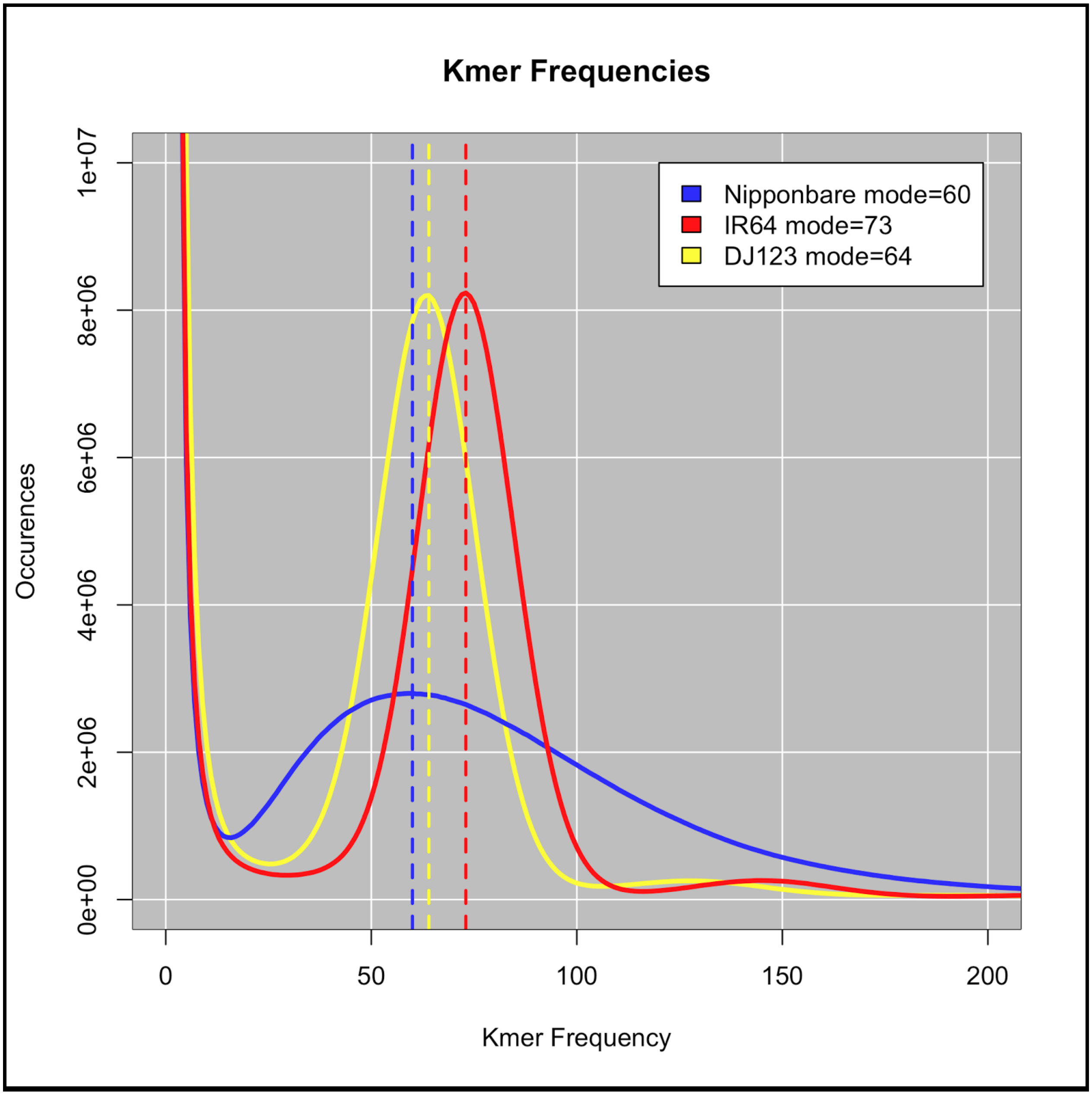

Supplementary Figure 1. K-mer frequency distributions for the three genomes.

The mode values of the distributions are highlighted as dashed vertical lines. DJ123 and IR64 have well resolved peaks from centered at their average coverage levels, Nipponbare has a broad peak because the library had the greatest proportion of duplicate reads.

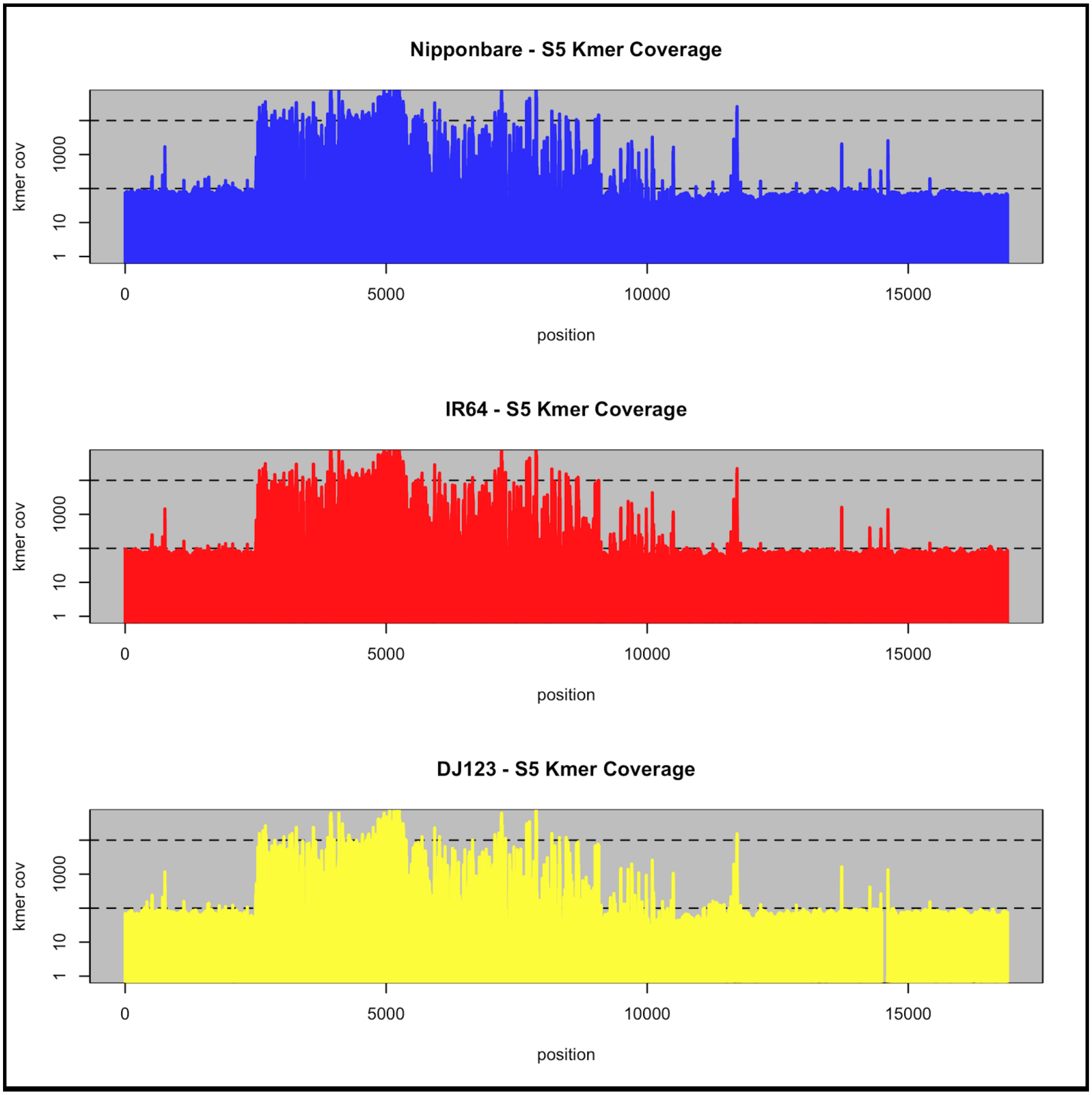

Supplementary Figure 2. K-mer coverage of the *S5* locus.

These plots display the k-mer coverage from the start of the CDS of ORF3 to the end of ORF5 of the Nipponbare reference sequence (NC_008399.2: 5744179-5761023). For clarity, 1x to 50,000x range (log scale) has been displayed in all the plots.

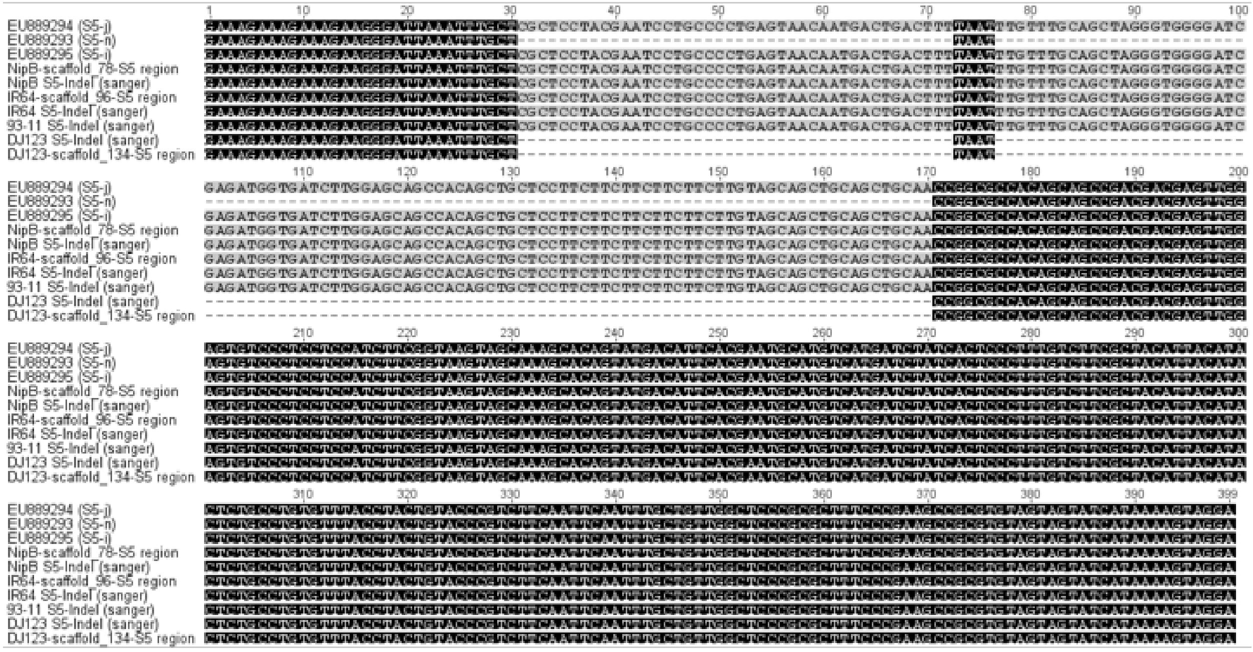

Supplementary Figure 3. Multiple alignment of *S5* Hybrid Sterility Locus: Indel Region

In particular, the indel predicted by the DJ123 scaffold was confirmed by Sanger sequencing.

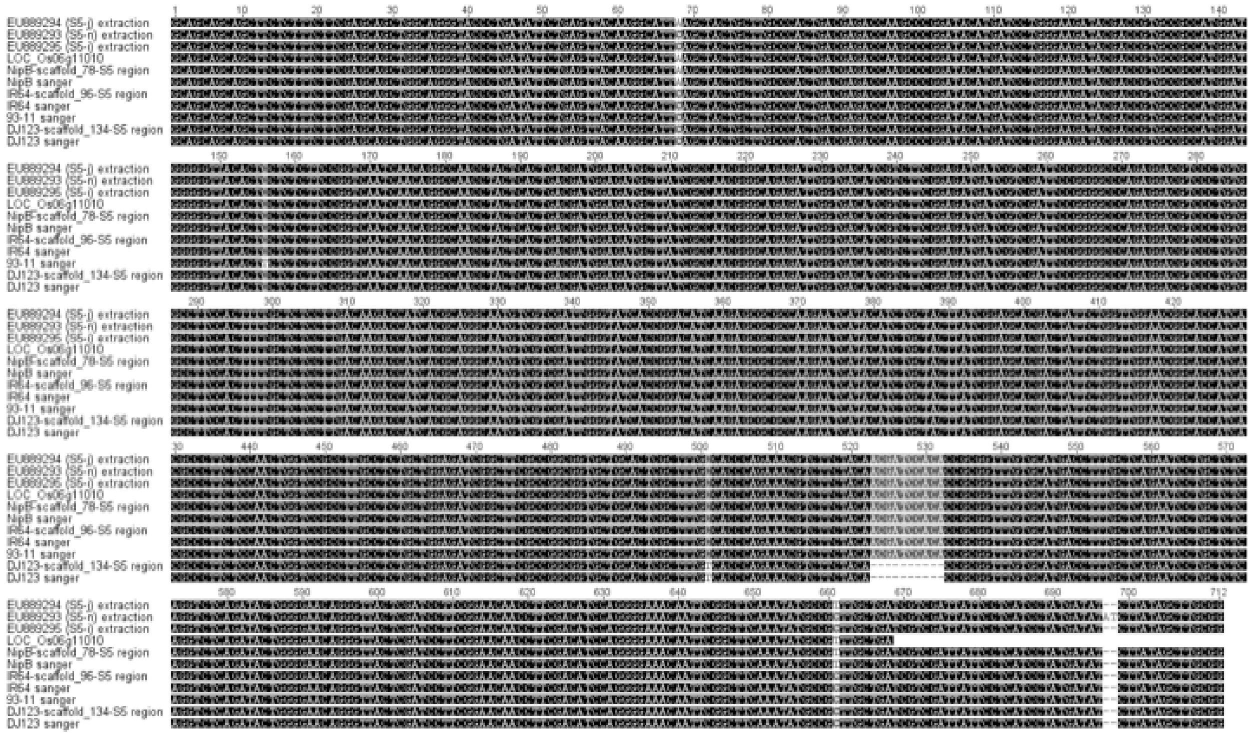

Supplementary Figure 4. Multiple alignment of *S5* Hybrid Sterility Locus: SNP region

In particular, the DJ123 scaffold shows a novel haplotype confirmed with Sanger sequencing.

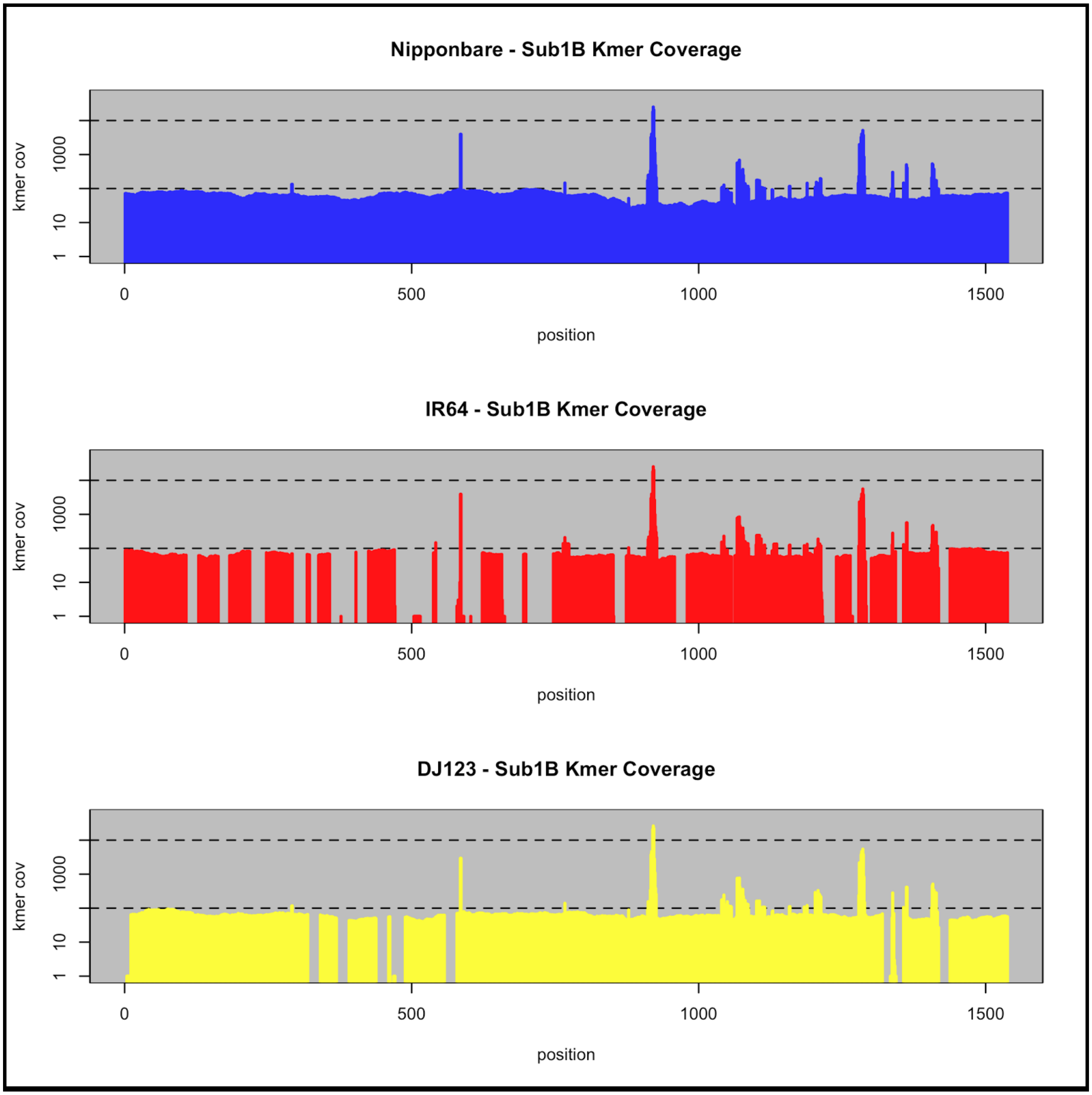

Supplementary Figure 5. K-mer coverage across the *Sub1B* gene.

For clarity, the range 1x to 50,000x (log scale) is displayed in all the plots.

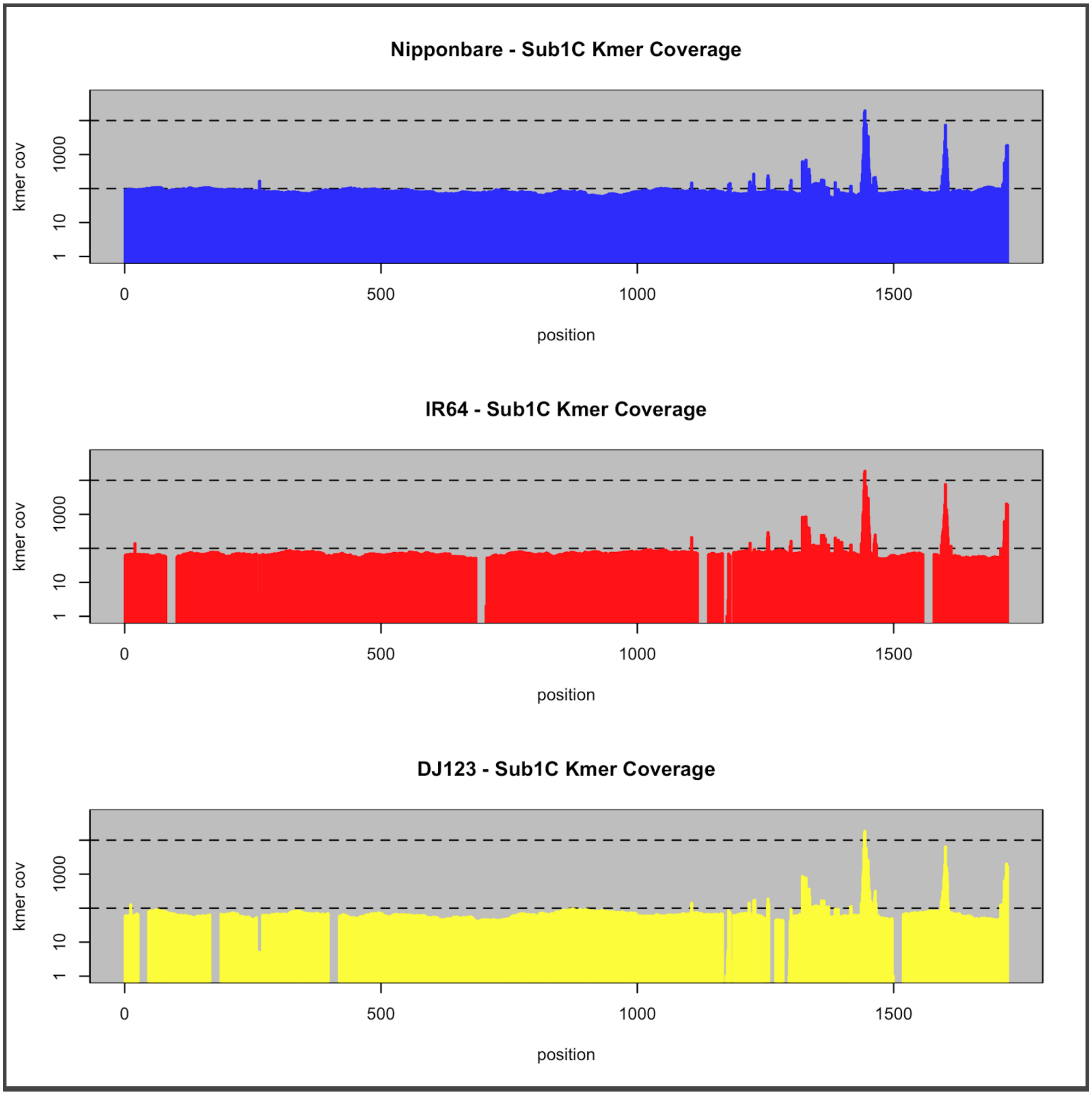

Supplementary Figure 6. K-mer coverage across the *Sub1C* gene.

For clarity, the range 1x to 50,000x (log scale) is displayed in all the plots.

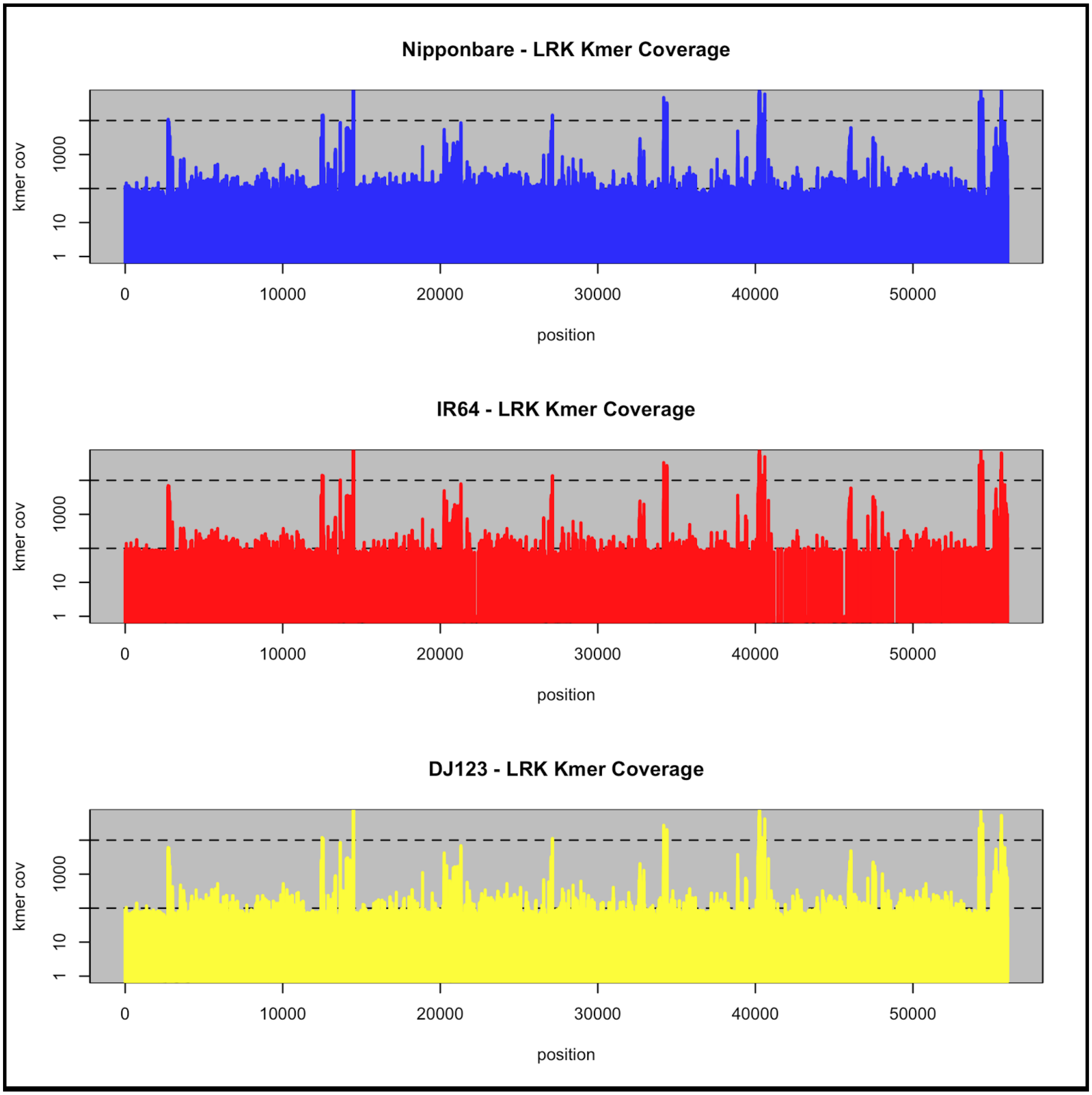

Supplementary Figure 7. K-mer coverage across the entire *LRK* region.

These plots display the k-mer coverage across the locus defined by the Nipponbare reference sequence (chr2:2930001-2986002). For clarity, the range 1x to 50,000x (log scale) is displayed in all the plots.

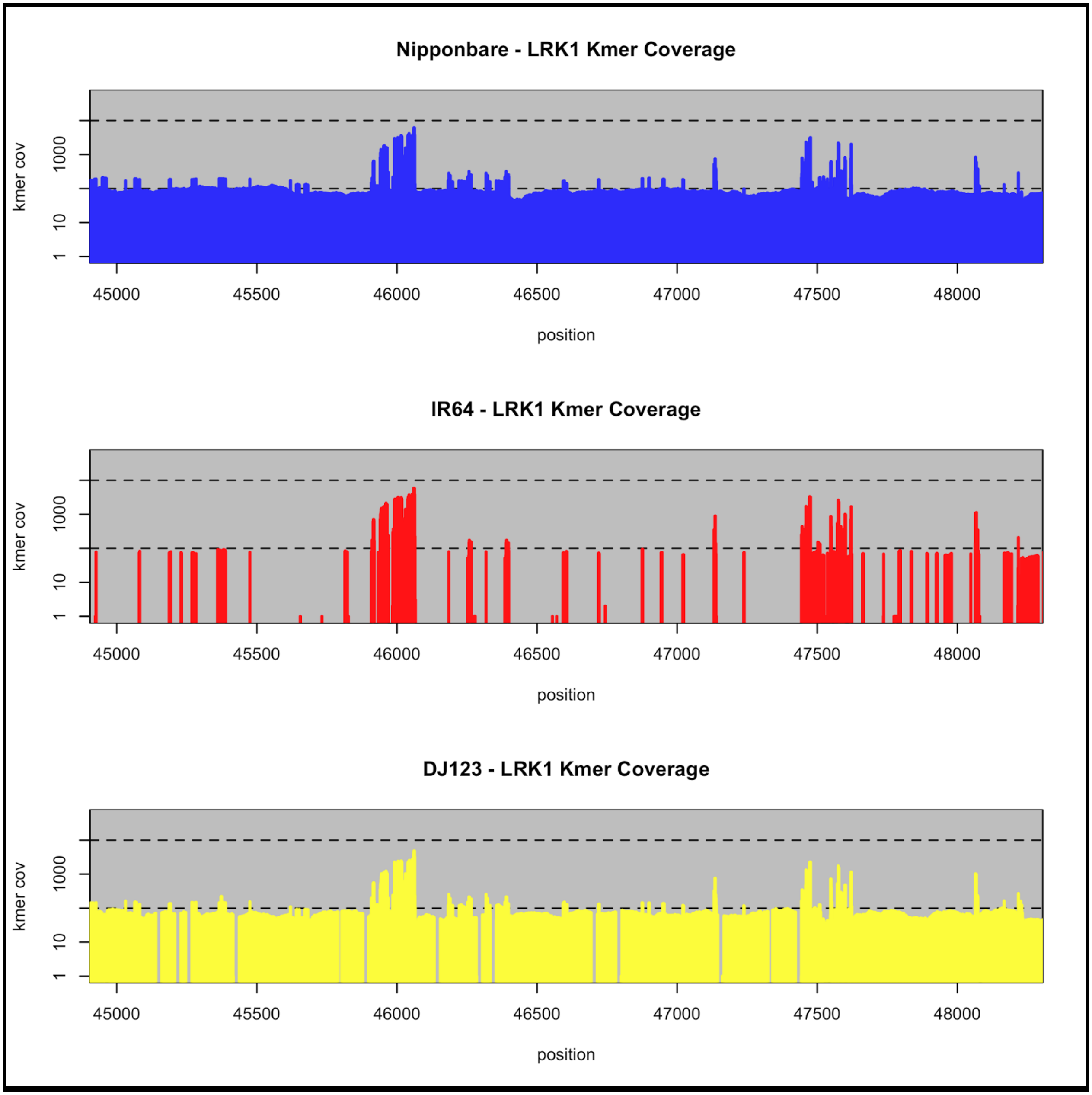

Supplementary Figure 8. K-mer coverage across the LRK1 gene

These plots display the k-mer coverage across the locus defined by the Nipponbare reference sequence (chr2:2930001-2986002) focusing on the interval for LRK1 (45030-48179 of this region). For clarity, the range 1x to 50,000x (log scale) is displayed in all the plots. Nipponbare and DJ123 have nearly uniform coverage across the gene except for SNPs in DJ123 shown as abrupt drops in coverage. The sparse coverage in IR64 indicates the presence of isolated k-mers and repeats shared with the other genomes, but an overall absence of the gene.

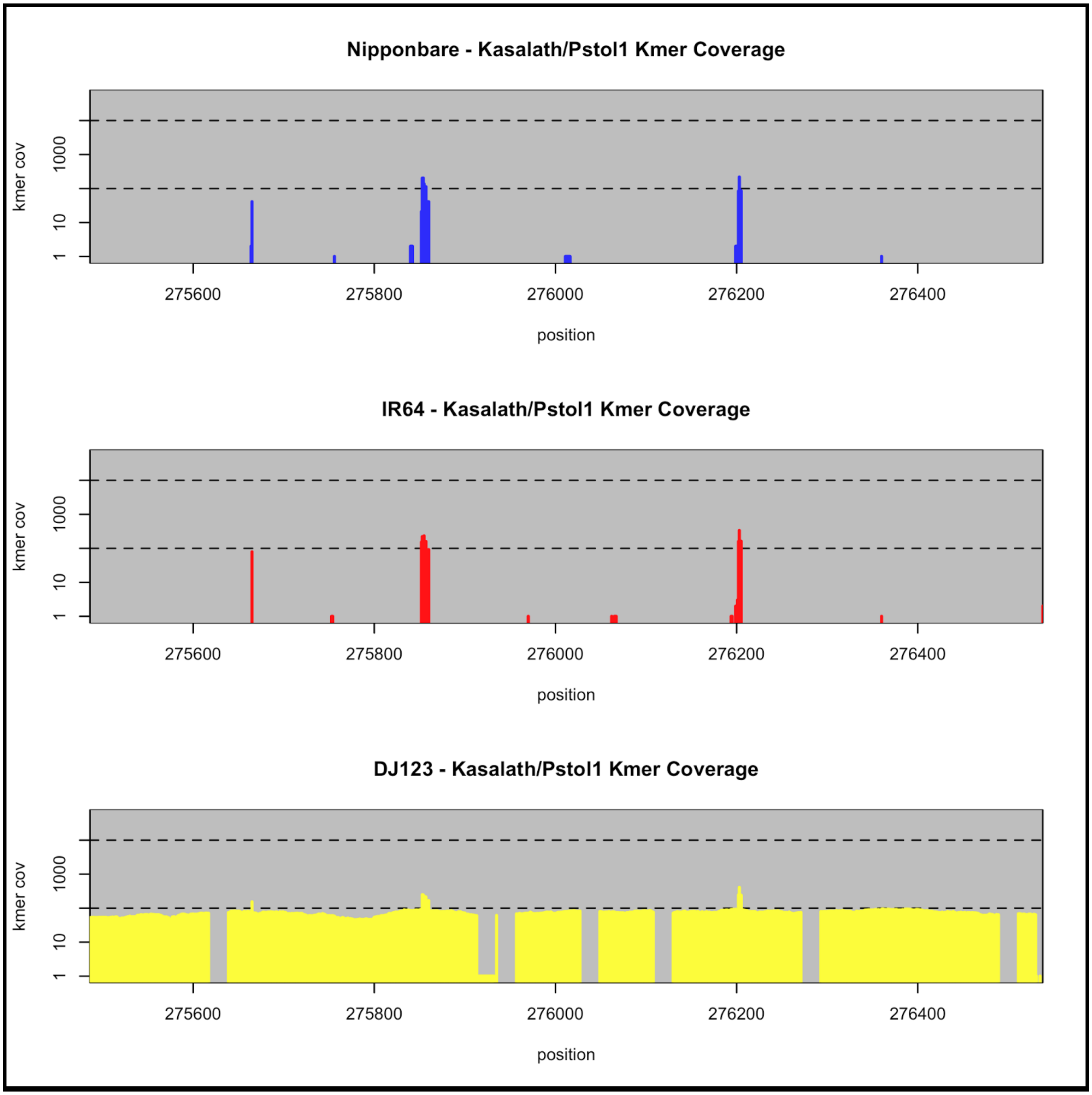

Supplementary Figure 9. K-mer coverage across the *Pstol1* gene

The k-mer coverage is plotted with respect to the reference Kasalath sequence (AB458444.1) across the *Pstol1* gene. For clarity, the range 1x to 50,000x (log scale) is displayed in all the plots.

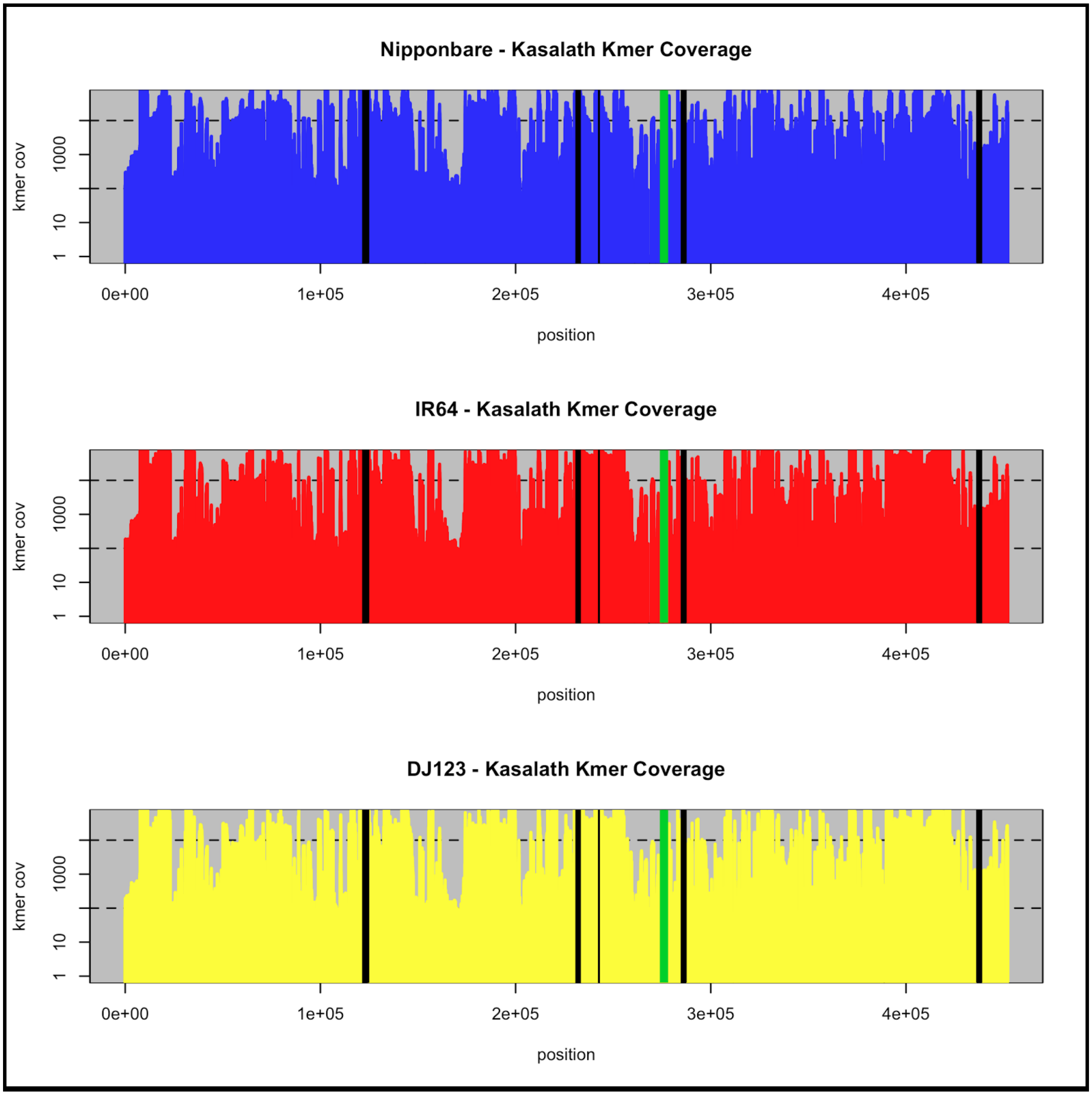

Supplementary Figure 10. K-mer coverage across the entire Pup1 region in Kasalath

The k-mer coverage is plotted with respect to the reference Kasalath sequence (AB458444.1) The total sequence span is 452 kbp. For clarity, the range 1x to 50,000x (log scale) is displayed in all the plots. Black vertical lines indicate gaps (Ns) in the reference sequence. The position of the *Pstol1* gene is highlighted in green. See Supplementary Figure 9 for just the coverage of *Pstol1*.

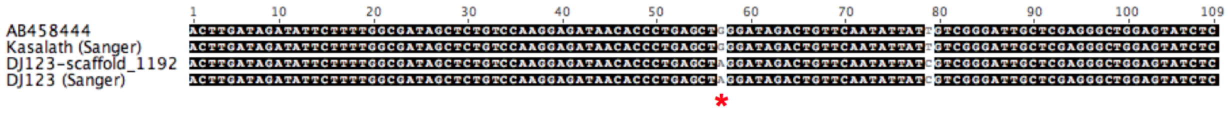

Supplementary Figure 11. Multiple alignment of *Pstol1* sequence fragment.

In particular, the SNP that introduces a premature stop codon in the DJ123 allele (red asterisk) was confirmed with Sanger sequencing.
